# Equity in the use of public services for mother and newborn child health care in Pakistan: a utilization incidence analysis

**DOI:** 10.1186/s12939-016-0405-x

**Published:** 2016-07-26

**Authors:** Sadia Mariam Malik, Nabila Ashraf

**Affiliations:** 1 1094 Vari Hall, Department of Economics, Faculty of Liberal Arts and Professional Studies, York University, 4700 Keele Street, Toronto, ON M3J 1P3 Canada; 2 Department of Economics, Quaid e Azam University, 45320 Islamabad, Pakistan

**Keywords:** Mother and newborn child health, Pakistan, Utilization Incidence Analysis, Public health spending

## Abstract

**Background:**

Poor maternal and infant health indicators are mostly concentrated among low income households in Pakistan and health care expenditures – especially on medical emergencies – are the most common income shocks experienced by the poor. Public investments in health are therefore considered as pro-poor interventions by the government of Pakistan. This study employs nationally representative household data for Pakistan for 2007–08 and 2010–11 to investigate whether benefits from publicly financed services on Mother and Newborn Child Health (MNCH) are effectively captured by the poor in terms of service utilization.

**Methods:**

The study conducts a *Utilization Incidence Analysis* of the use of public health services for MNCH in Pakistan. For this purpose, the utilization shares of households, ranked by economic status, are computed. The concentration curves are plotted and their dominance is tested against an equal distribution and Lorenz curves to determine whether the distribution is pro-poor and progressive.

**Results:**

Although the shares of bottom income groups in the utilization of most services for MNCH have increased between 2007 and 2011, the utilization of some services such as post-natal consultation; institutional maternal delivery; and Tetanus Toxoid injections for pregnant women remains pro-rich in 2011. The utilization of pre-natal consultation, especially through lady health workers and visitors; the use of Family Panning Units; and immunization services is somewhat evenly distributed. The use of Basic Health Units (BHUs) is found to be pro-poor. The provincial analysis reveals that the province of Baluchistan depicts an unusually high level of inequity in the distribution of utilization benefits from almost all public health services. Finally, in terms of progressivity, public spending on all health services analyzed in the study is found to be progressive at the national level implying that investment in MNCH has the potential to redistribute income from rich to the poor.

**Conclusion:**

To target the poor effectively, the study recommends expanding the network of BHUs as well as basic reproductive and child health care services. The outreach of health facilities in Baluchistan need to be expanded while targeting the poor effectively by mitigating various access costs that prevent them from using public health services.

**Electronic supplementary material:**

The online version of this article (doi:10.1186/s12939-016-0405-x) contains supplementary material, which is available to authorized users.

## Background

Pakistan, a lower middle income country, with an estimated population of about 185 million [1], faces considerable challenges in health particularly that pertaining to mothers, infants, and children. Poor maternal and child health indicators are often accompanied by widening socioeconomic and regional disparities. Nearly half of the total births are not attended by skilled health personnel [2]. The neonatal mortality rate at 42 per 1,000 live births [3] is one of the highest in the world. Child malnutrition is extremely high and is worsening or stagnant at best, over time [4]. The country has also been lagging in terms of achieving the Millennium Development Goals (MDG) related to maternal and child health indicators [5].

In terms of disparities in health, infant mortality rate is 94 per 1000 live births, among households belonging to poorest wealth quintile compared to 53 among those falling into the richest wealth quintile [6]. At the regional level, maternal mortality ratio varies from 785 in the province of Baluchistan to 227 per 100,000 live births, in the province of Punjab [6]. Even within a particular province such as Punjab, infant mortality rate varies from a high of 110 in the district of Bahawalpur to 46, per 1,000 live births in the district of Rawalpindi [7]. Rural urban disparities are also prominently high. In Punjab for instance, child mortality rate varies from 126 in rural areas to 76 per 1000 live births in urban areas [7]. These inequities in the heath of infants and children are deemed unfair as they can lead to a life time denial of opportunities and capabilities and can undermine the important goal of achieving equality of opportunities.

Much of these inequities in maternal and new-born child health indicators can be explained by inequity in the utilization of health care services. The Pakistan Demographic and Health Survey (PDHS) indicates that only 37 % of the women from bottom wealth quintile^1^ receive prenatal care from a skilled health care provider compared to 92 % of the women in the top wealth quintile who receive such care [6]. Similarly, only 12.4 % of the women in bottom quintile (lowest 20 % income earners) have their delivery in a health facility compared to 73.8 % of the corresponding women in top wealth quintile [6].

In countries like Pakistan, where insurance and social security arrangements are almost non-existent and households rely almost exclusively on out of pocket expenditures to pay for their medical care,^2^ the concentration of poor health indicators among low income groups adds to the vulnerability of these households. In particular, households whose incomes are just above the poverty line, can fall below the poverty line as a result of an accident or a sudden illness. In fact, evidence suggests that health care expenditures related to medical emergencies are found to be the most common source of economic shocks faced by households in Pakistan^3^ [8].

Like many other developing countries that lack the fiscal space required to run a full-fledged program of social security and poverty reduction through cash transfers, public spending on health in Pakistan is seen as an instrument to reduce poverty and promote an equitable distribution of income. The health sector allocations have often been monitored and reported under the country’s Poverty Reduction Strategy Programme (PRSP). A number of flagship programmes in the area of basic and preventive health care in particular, are currently being run by the government of Pakistan. These include the Lady Health Workers Programme; Expanded Program of Immunization; Malaria Control Programme; Tuberculosis and HIV/AIDS Control Programme; National Maternal and Child Health Programme; and Food and Nutrition Programme etc. [5].

Little is known however about the effectiveness with which these programmes and public health services are targeted towards the poor and whether or not the poor actually benefit from them. The objective of the present study is to investigate whether benefits from public spending on Mother, Newborn, and Child Health (MNCH) care services, in particular, are effectively utilized by the poor in Pakistan.

## Methods

As mentioned earlier, the present study employs household data from Pakistan Social and Living Standards Measurement Survey (PSLM) for 2007–08 and 2010–11 to investigate whether benefits from public spending on health are effectively captured by the poor in terms of service utilization. More specifically, the study focuses on the most vulnerable group comprising of mothers, newborn, and children and answers the following questions: Which income groups benefit more in terms of utilizing MNCH related public health services in Pakistan? Which services are more pro-poor/pro-rich than others? Which provinces depict greater inequality in the distribution of benefits from public spending on MNCH? What is the observed trend in the distribution of utilization benefits of MNCH related public services in Pakistan between 2007–08 and 2010-11? And finally what is the likely impact of public health spending on MNCH on the redistribution of living standards in Pakistan?

To answer these questions, we use the ‘Utilization Incidence Approach’ also referred to as the ‘Participation Incidence Approach.’ A number of studies have applied this approach, particularly in the context of examining benefits from maternal and child health care services [9–14]. The present study is the first ever attempt to apply this approach in the context of Pakistan - particularly at the subnational level – to analyse the socio-economic distribution of the utilization of MNCH related public health services.

Following this approach, we first rank all respondents who had used a public health facility or service for their last pregnancy, according to their living standards as measured by their consumption expenditures. We then estimate how the usage of these services is distributed across socioeconomic groups. Simple binary indicator codes 1 if a particular service is used and 0 otherwise. An important assumption here is that all those who use a particular service or participate in a programme receive the same benefit. We then compare the utilization shares of each socioeconomic group with its share in total population so as to investigate whether the utilization of public health services is pro-poor or pro-rich. If for example, the share of poorest 20 % of the population is more than its share in total population (which is 20 %), then the utilization incidence is pro-poor.

The incidence of public health services utilization is also described by its concentration curve that plots the cumulative percentage of the health variable on y-axis against the cumulative percentage of the population, ranked by living standards (from poorest to the richest) on x-axis [15]. The 45° line of equality represents perfect equality in the distribution of utilization benefits. If the concentration curve coincides with the line of equality, the utilization benefits are equally distributed across income quintiles. If the concentration curve lies above the 45° line, public health services utilization is relatively more concentrated amongst the poor income groups. The concentration curve in this case is said to dominate the 45° line of equality and the distribution is said to be pro-poor. If on the other hand, concentration curve lies below the 45° line (45° line dominates the concentration curve), the utilization of public health services is regarded as pro-rich.

If benefits from public health services are considered as part of final income, then one might be interested in examining whether public health subsidies reduce income inequality or not. If benefits from public health services utilization are more equally distributed than the distribution of income/consumption, then utilization of public health services is thought to reduce income inequality and is referred to as progressive. In this particular case, the concentration curve lies above (or dominates) the Lorenz curve. If on the other hand, public health services utilization is distributed more unequally than consumption (i.e. the concentration curve lies below the Lorenz curve or Lorenz curve dominate the concentration curve of benefits), services are said to be regressive [14, 15].

In order to test whether the concentration curve dominates the 45° line or the Lorenz curve, dominance testing is conducted by comparing the concentration curves of utilization benefits with two bench marks that are 45° line and Lorenz curve. We use the standard decision rule which is to reject the null of non-dominance in favour of dominance if there is at least one significant difference between curves (or a curve and the 45° line) in one direction and no significant difference in the other direction across 19 equally spaced quantile points [15].

In order to compare the *magnitude* of the extent of inequality between 2007–08 and 2010–11 and between provinces, the information embodied in concentration curves is inadequate. For this purpose, we compute the concentration indices that are a summary measure of the magnitude of inequality and are defined as twice the area below the 45° line and the concentration curve. The value of concentration index lie between −1 (poorest quantile utilizes all benefits) and +1 (richest quantile utilizes all benefits). The value of concentration index is zero if there is no socio-economic inequality.

### Data and variables

The micro-level household data sets of Pakistan Social and Living Standards Measurement Survey (PSLM) for 2007–08 and 2010–11 along with the accompanying Household Integrated Economic Survey (HIES) are utilized for the purpose of our analysis. These household surveys are representative at the national, provincial, and district level. PSLM collects data on key social indicators such as education, health, and population welfare etc. whereas HIES collects data on consumption expenditures and income. The HIES module is used together with that of PSLM and the two surveys are integrated with each other.^4^ The HIES/PSLM surveys that are representative at national *as well as* provincial level are conducted after an interval of two years. Since our study involves provincial level analysis, the two consecutive surveys, representative at both provincial as well as national level at the time of our study were 2007–08 and 2010–11. Since these two surveys which were held in different time periods, have the same questionnaire as well as the same survey methodology, the results can be used to analyze the trend between 2007 and 2011 with a fair degree of accuracy. The PSLM reports that are published by the government of Pakistan carry out such trend analysis over time [16].

Data on all variables related to public health services for MNCH is utilized from the surveys. These include pre-natal consultation; postnatal consultation; maternal delivery; Tetanus Toxoid injections; and child immunization services. Since the focus of present study is on public provision of health care facilities, the health care provider codes of Lady Health Workers (LHW), Lady Health Visitors, Govt. hospitals/Clinics, Family Welfare Centers, Reproductive Health Service Units and Mobile Service Units are considered as public providers for prenatal and postnatal consultation. For child delivery, government hospital/clinic is considered as public provider. Child immunization services are almost exclusively provided by public sector health care delivery system under the Expanded Programme of Immunization.

A distinguishing feature of PSLM 2010–11 - that sets it apart from previous surveys - is that it includes a separate module on benefit from services and facilities. The health related services included in this module are Basic Health Unit (BHU) and Family Planning Unit (FPU). These two variables are also analyzed for their usage by income groups.

In keeping with the generally accepted convention, the living standards indicator that we use is consumption expenditure. More precisely, we use ‘monthly per Adult Equivalent Consumption Expenditure.’ A relatively simple equivalence scale: 0.8 for all those household members younger than 18 years and 1 for all other household members is used for per adult measurement.

## Results

Before presenting the results on Utilization Incidence Analysis, it is pertinent to look at the key socioeconomic characteristics of survey respondents, as presented in Table 1. The average household size of the respondents consists of about six to seven members. Average monthly consumption expenditure per capita was Pk. Rs. 1923 (around U.S. $20 unadjusted for purchasing power) in 2007–08 that increased to Pk. Rs. 3029 (about U.S.$30, unadjusted for purchasing power) in 2010–11. The literacy rate, defined as the percentage of respondents (10 years and older) who can read and write, is about 58 % for both sexes and 46 % for females. The infant mortality rate, a summary measure of the health of children, is around 69 per 1000 live births.

**Table 1:** Key characteristics of survey respondents

	2007-08	2010-11
*Key Social Indicators*		
* Average household size*	6.58	6.38
* Average monthly consumption expenditure per capita (Pk. Rs.)*	1923	3029
* Literacy rate (% total)*	56	58
* Literacy rate (% female)*	44	46
* Infant mortality rate (per 1000, live births)*	69	N.A

Source: Reports published by Pakistan Bureau of Statistics on a) Household Integrated Economic Survey of Pakistan: 2007–08 and 2010–11

b) Pakistan Living Standards Measurement Survey: 2007–08 and 2010–11. Retrieved on June 6, 2016 from www.pbs.gov.pk

### National level results

We begin by analysing the utilization shares of public health services for MNCH at the national level in 2007–08 and 2010–11. These results, categorized by the type of service/facility, are presented in Table 2. The results show that in 2007–08, the utilization incidence of all public health services was pro-rich with the utilization shares of the richest income quintiles exceeding their population shares. The usage of postnatal consultation was most pro-rich followed by institutional delivery with the richest 20 % of the population having a share of 36 % and 32 % in the total utilization of postnatal consultation and institutional maternal delivery respectively. The respective shares of the poorest 20 % in the utilization of these services were merely 12 % for postnatal consultation and 13 % for institutional delivery.

**Table 2:** The utilization incidence of public services for MNCH in Pakistan

	2007-08						2010-11					
	Quintiles						Quintiles					
	Poorest	II	III	IV	V	Total	Poorest	II	III	IV	V	Total
*Type of Service/Facility (%)*												
* Prenatal Consultation*	0.16	0.20	0.19	0.22	0.23	1.00	0.21	0.22	0.20	0.22	0.16	1.00
* Hospital based Maternal Delivery*	0.13	0.16	0.16	0.23	0.32	1.00	0.15	0.21	0.21	0.23	0.21	1.00
* Postnatal Consultation*	0.12	0.12	0.17	0.23	0.36	1.00	0.15	0.21	0.19	0.23	0.23	1.00
* Tetanus Toxoid injections*	0.15	0.18	0.19	0.22	0.26	1.00	0.15	0.17	0.19	0.23	0.27	1.00
* Immunization Services*	0.17	0.19	0.19	0.21	0.24	1.00	0.18	0.19	0.19	0.21	0.22	1.00
* Basic Health Units*							0.26	0.25	0.21	0.18	0.11	1.00
* Family Planning Services*							0.25	0.18	0.19	0.19	0.18	1.00

**Fig. 1:**
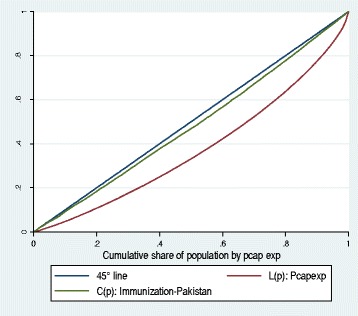
The Utilization Incidence of Immunization Services in Pakistan: Concentration Curve and Lorenz Curve in Pakistan (2010–11)

Although the utilization shares of bottom income quintile went up for all services in 2010–11, these shares remain significantly below their population shares for many services such as institutional delivery, postnatal consultation, and Tetanus Toxoid injections for pregnant women. The utilization incidence of immunization services appears to be slightly pro-rich with the shares of the poorest and richest income quintile being 18 and 22 % respectively. The concentration curve for immunization services for 2010–11 (presented in Fig. 1) lies below, yet quite closely to, the 45° line of equality indicating a somewhat proportionate distribution of utilization benefits.

However, the results of tests of dominance of concentration curve for immunization services against the 45° line, presented in Table 3, indicate that the 45° line dominates the concentration curve implying that the incidence of immunization services is pro-rich although the magnitude of inequality as depicted by the concentration index (refer to Table 4) is quite low at 0.04.

**Table 3:** Tests of dominance of concentration curves for public health services use in Pakistan

	2007-08		2010-11	
Type of Service/Facility	45°	Lorenz	45°	Lorenz
Prenatal Consultation	-	+	+	+
Hospital based Maternal Delivery	-	+	-	+
Postnatal Consultation	-		-	+
Tetanus Toxoid injections	-	+	-	+
Immunization Services	-	+	-	+
Basic Health Units			+	+
Family Planning Services				+

Note: − indicate the 45° line/Lorenz curve dominates the Concentration curve. + indicate Concentration curve dominates 45° line/Lorenz curve. Blank cell indicates failure to reject the null hypothesis that curves are indistinguishable using the multiple comparison test. Dominance is rejected if there is at least one significant difference in one direction and no significant difference in the other, with comparisons at 19 quintiles and 5 % significant level

**Table 4:** Concentration indices for the utilization of public health services for MNCH in Pakistan

	2007-08	2010-11
Prenatal Consultation	0.06	−0.05
Hospital based Maternal Delivery	0.19	0.05
Postnatal Consultation	0.26	0.07
Tetanus Toxoid injections	0.11	0.14
Immunization Services	0.06	0.04
Basic Health Units		−0.15
Family Planning Services		−0.05

Note: Concentration indices lie between −1 and +1. Negative values indicate pro-poor incidence and positive values indicate pro-rich incidence

For other services such as institutional maternal delivery; postnatal consultation; and Tetanus Toxoid injections, the tests of dominance conclude pro-rich incidence in 2010–11. The concentration indices for these services, presented in Table 4, are all positive indicating a pro-rich incidence although the extent of inequality has been reduced significantly between 2007–08 and 2010–11.

**Fig. 2:**
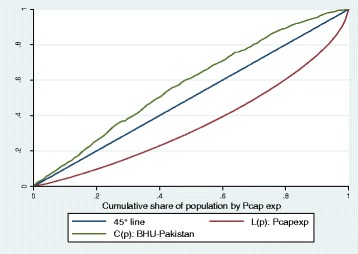
The Utilization Incidence of Basic Health Units (BHUs) in Pakistan: Concentration Curve and Lorenz Curve

Table 4 shows that in 2007–08, the utilization incidence of postnatal consultation was most pro-rich, whereas in 2010–11, it is the Tetanus Toxoid injections for pregnant women that appear to be most pro-rich. The utilization incidence of Basic Health Units (BHUs) appears to be the most pro-poor with concentration index of −0.15 and the concentration curve dominating the 45° line (refer to Table 3 and Fig. 2).

Data on utilization share for Basic Health Units (BHUs) also show a clear pro-poor orientation with the utilization share of the poorest income quintile being 26 % against 11 % for the richest quintile (refer to Table 2).

The use of Family Planning Units (FPUs) also show a pro-poor orientation as far as the utilization shares are concerned with the share of the poorest income quintile being 25 % against 18 % for the richest income quintile. However, the tests of dominance do not allow us to reject the null hypothesis that the concentration curve and line of equality are indistinguishable thereby indicating a proportionate distribution (refer to Table 3).

With respect to progressivity, public health services utilization is considered to be progressive if the distribution of benefits in terms of utilization is more equal than the distribution of consumption. In this case, the concentration curve for utilization benefits should lie everywhere above the Lorenz curve for consumption or the concentration curve should dominate the Lorenz curve. The results of dominance tests presented in Table 3 indicate the dominance of concentration curve over Lorenz curve for all services implying that public health spending on all MNCH related services analysed in the study has the potential to redistribute economic welfare from rich to the poor.

In Table 5, we present the concentration indices for prenatal and postnatal consultation by type of provider. More specifically, we are interested in analyzing the utilization of door to door service provided by one of the largest and reportedly one of the most effective primary health care programmes in Pakistan: The Lady Health Workers Programme [17], and compare its usage with that of hospitals or Rural Health Centres (RHC). For this purpose, we isolate the type of public provider for these consultations into two categories: 1) public hospital/Rural Health centre (RHC) and 2) Lady Health Worker (LHW)/Lady Health Visitors (LHV). The estimation results show that in 2007–08, the utilization incidence of both prenatal and postnatal consultation was pro-rich irrespective of the type of health service provider with the extent of inequality higher if the service was provided in a health care facility such as hospital or Rural Health Centre. In 2010–11, the utilization incidence became proportionate for prenatal consultation provided in hospital based facility and pro-poor for the consultation provided by Lady Health Workers (LHWs) or Lady Health Visitor (LHVs). The incidence of postnatal consultation, on the other hand is pro-rich if the consultation is provided by a public hospital, RHC or BHU. However, the incidence turns from pro-rich to pro-poor if the type of provider changes from hospital based facility to LHW/LHVs. The plausible reasons for this could be the reduction in access costs, especially those related to transportation, if the service is provided by a community health worker or a health visitor. This is an important finding suggesting the effectiveness of LHVs and LHWs in expanding access to reproductive health facilities particularly among the low income groups.

**Table 5:** Concentration indices for prenatal and postnatal consultation by type of health care provider (All Pakistan)

	2007-08		2010-11	
Year	Public Hospital/RHC	LHV/LHW	Public Hospital/RHC	LHV/LHW
2007-08	0.068	0.050	0.298	0.192
2010-11	−0.031	−0.110	0.109	−0.026

Note: *RHC* stands for Rural Health Centre, *LHV* stands for Lady Health Visitor and *LHW* stands for Lady Health Worker

Concentration indices lie between −1 and +1. Negative values indicate pro-poor incidence and positive values indicate pro-rich incidence

### Provincial level results

Since the provision of health services in Pakistan have lately been devolved from federal to the provincial level [18, 19], it is useful from a policy perspective, to analyze where each province stands in terms of equity in the use of public health services. There are four provinces in Pakistan: Punjab, Sindh, Baluchistan and Khyber Pakhtunkhwa (KPK). These provinces are quite diverse not only in terms of their geographic and ecological characteristics but also in terms of socioeconomic characteristics. The provincial level results, presented in Tables 6, 7 and 8, show that although these results broadly follow the pattern observed at the national level, there are quite a few important exceptions. For instance, between 2007 and 2011, the socioeconomic distribution of utilization improved for all services except the Tetanus Toxoid injections for pregnant women that remained the same in Punjab and worsened in all other provinces (refer to Table 6).

**Table 6:** The utilization incidence of public services for MNCH at the Provincial level in Pakistan

	2007-08						2010-11					
	Quintiles						Quintiles					
	Poorest	II	III	IV	V	Total	Poorest	II	III	IV	V	Total
*Type of Service/Facility (%)*												
Punjab												
* Prenatal Consultation*	0.16	0.16	0.18	0.24	0.26	1.00	0.22	0.24	0.22	0.19	0.13	1.00
* Hospital based Maternal Delivery*	0.09	0.10	0.15	0.28	0.38	1.00	0.12	0.24	0.22	0.20	0.22	1.00
* Postnatal Consultation*	0.10	0.10	0.14	0.25	0.40	1.00	0.12	0.20	0.21	0.23	0.24	1.00
* Tetanus Toxoid injections*	0.15	0.18	0.20	0.23	0.25	1.00	0.15	0.18	0.19	0.22	0.25	1.00
* Immunization Services*	0.17	0.19	0.20	0.21	0.23	1.00	0.19	0.19	0.19	0.21	0.22	1.00
* Basic Health Units*							0.27	0.24	0.20	0.18	0.11	1.00
* Family Planning Services*							0.23	0.17	0.20	0.20	0.20	1.00
Sindh												
* Prenatal Consultation*	0.15	0.22	0.21	0.24	0.17	1.00	0.19	0.24	0.17	0.25	0.16	1.00
* Hospital based Maternal Delivery*	0.15	0.22	0.19	0.22	0.23	1.00	0.19	0.20	0.22	0.27	0.12	1.00
* Postnatal Consultation*	0.12	0.13	0.20	0.25	0.30	1.00	0.26	0.13	0.20	0.26	0.15	1.00
* Tetanus Toxoid injections*	0.18	0.17	0.18	0.20	0.28	1.00	0.11	0.17	0.18	0.25	0.29	1.00
* Immunization Services*	0.16	0.17	0.20	0.21	0.26	1.00	0.18	0.19	0.19	0.22	0.23	1.00
* Basic Health Units*							0.12	0.28	0.26	0.24	0.11	1.00
* Family Planning Services*							0.10	0.13	0.30	0.19	0.29	1.00
Khyber Pakhtunkhwa												
* Prenatal Consultation*	0.17	0.21	0.20	0.23	0.19	1.00	0.19	0.19	0.18	0.20	0.23	1.00
* Hospital based Maternal Delivery*	0.18	0.22	0.19	0.18	0.23	1.00	0.18	0.20	0.20	0.22	0.20	1.00
* Postnatal Consultation*	0.17	0.12	0.21	0.19	0.31	1.00	0.16	0.27	0.17	0.18	0.22	1.00
* Tetanus Toxoid injections*	0.20	0.20	0.19	0.18	0.23	1.00	0.16	0.15	0.19	0.24	0.27	1.00
* Immunization Services*	0.21	0.21	0.20	0.18	0.20	1.00	0.19	0.20	0.20	0.20	0.21	1.00
* Basic Health Units*							0.28	0.26	0.20	0.15	0.10	1.00
* Family Planning Services*							0.26	0.26	0.14	0.22	0.13	1.00
Baluchistan												
* Prenatal Consultation*	0.13	0.20	0.18	0.19	0.30	1.00	0.14	0.22	0.19	0.22	0.24	1.00
* Hospital based Maternal Delivery*	0.20	0.14	0.08	0.14	0.44	1.00	0.12	0.17	0.20	0.20	0.32	1.00
* Postnatal Consultation*	0.17	0.06	0.18	0.15	0.45	1.00	0.11	0.15	0.16	0.24	0.35	1.00
* Tetanus Toxoid injections*	0.10	0.16	0.19	0.19	0.36	1.00	0.07	0.13	0.17	0.23	0.40	1.00
* Immunization Services*	0.19	0.19	0.19	0.20	0.23	1.00	0.15	0.20	0.17	0.22	0.27	1.00
* Basic Health Units*							0.15	0.21	0.22	0.22	0.20	1.00
* Family Planning Services*							0.30	0.26	0.19	0.08	0.18	1.00

**Table 7:** Concentration Indices for the utilization of public health services for MNCH at the Provincial Level in Pakistan (2010–11)

Type of Service/Facility	Punjab	Sindh	Khyber Pakhtunkhwa	Baluchistan
Prenatal Consultation	−0.09	−0.03	0.04	0.08
Hospital based Maternal Delivery	0.08	−0.04	0.03	0.18
Postnatal Consultation	0.11	−0.03	0.01	0.24
Tetanus Toxoid injections	0.10	0.18	0.13	0.32
Immunization Services	0.03	0.05	0.01	0.11
Basic Health Units	−0.16	−0.01	−0.20	0.03
Family Planning Services	0.01	0.19	−0.13	−0.16

Note: Negative values indicate pro-poor incidence and positive values indicate pro-rich incidence

**Table 8:** Tests of Dominance of Concentration Curves for Public Health Services Use at the Provincial Level in Pakistan (2010–11)

	Punjab		Sindh		Khyber Pakhtunkhwa		Baluchistan	
Type of Service/Facility	45°	Lorenz	45°	Lorenz	45°	Lorenz	45°	Lorenz
Prenatal Consultation	+	+	+	+		+		+
Hospital based Maternal Delivery	-	+	+	+		+	-	
Postnatal Consultation	-	+		+		+	-	
Tetanus Toxoid injections	-	+	-	+	-	+	-	-
Immunization Services	-	+	-	+		+	-	+
Basic Health Units	+	+	-	+	+	+		+
Family Planning Services		+	-	-		+		+

Note: − indicate the 45° line/Lorenz curve dominates the Concentration curve. + indicate Concentration curve dominates 45° line/Lorenz curve. Blank cell indicates failure to reject the null hypotheis that curves are indistinguishable using the multiple comparison test. Dominance is rejected if there is at least one significant difference in one direction and no significant difference in the other, with comparisons at 19 quintiles and 5 % significant level

For all other services, the utilization shares of the bottom income quintile increased over time. Notwithstanding this improvement, the shares of the bottom income quintiles in the utilization of majority of the services such as hospital based maternal delivery; postnatal consultation; Tetanus Toxoid injections; and immunization services still remain below their respective population shares. The utilization shares of the bottom income quintile are especially low for all these services in the province of Baluchistan.

In fact, Baluchistan stands out from the rest of the provinces in the sense that the utilization incidence of all services except Family Planning Units (FPU) remains highly pro-rich in this province. The use of BHUs which is clearly found to be pro-poor at the national level as well as in Punjab and KPK is found to be pro-rich in Baluchistan. The concentration indices, reported in Table 7 show that the usage of all services (with the exception of Family Planning Units) in Baluchistan are positive (indicating a pro-rich incidence) and the size of these indices are significantly higher compared to other provinces. Interestingly, the use of Family Planning Units is found to be pro-poor in Baluchistan with the poorest income quintile having a share of 30 % in the total utilization of these services against 18 % for the richest quintile.

The use of Family Planning Units in Punjab and KPK is somewhat proportionately distributed: the concentration curves and the 45° lines of equality for these provinces are indistinguishable according to the result of multiple comparison test reported in Table 8. In Sindh however, the incidence of usage of Family Planning Units is pro-rich (The concentration curves for all services at the national as well as provincial level are provided in Additional file 1 for those interested in more details).

With regards to progressivity, our analysis show that the public provision of all health services analysed in the study is inequality reducing across all provinces except Baluchistan where the provision of some services such as institutional maternal delivery and postnatal consultation is neither progressive nor regressive while the use of Tetanus Toxoid injections is regressive (refer to Table 8).

## Discussion

### Key findings

Our analysis above yields some interesting results. *First*, we find that the utilization shares of the bottom income quintile improved over time and turned from pro-rich in all services in 2007 to pro-poor in many services in 2011. This is a positive trend and could be due to the country’s transition to democracy in 2007 that resulted in the expansion of some publicly funded basic health care programmes like the National Programme for Family Planning and Primary Health Care; Expanded Programme of Immunization; Lady Health Worker Programme; and National Maternal Neonatal and Child Health (MNCH) Programme [5].

*Second,* we find that the utilization incidence of some services such as post-natal consultation; hospital based maternal delivery; and tetanus toxoid injection for pregnant women, remains pro-rich in 2011. This could be due to both demand side factors such as the inability to pay for access costs - that may include transportation costs, user fees, or the opportunity cost in terms of foregone income - or due to supply side factors such as inadequate availability of emergency obstetric care especially in remote rural areas where majority of the poor reside.

*Third*, we find that the utilization of services that fall within the purview of basic health services such as immunization; prenatal consultation especially through Lady Health Workers (LHWs) and lady Health Visitors (LHVs); and Basic Health Units (BHUs) is either pro-poor or more equitably distributed than specialized services such as hospital based maternal delivery and post-natal consultation. This is consistent with the evidence obtained in the context of other developing countries where the benefits of hospital based services are often captured by the rich rather than the poor [20].

*Fourth*, at the provincial level, we find that the extent of inequality in the distribution of utilization benefits is much higher in Baluchistan compared to the rest of the three provinces. *Finally*, in terms of progressivity, our findings indicate that the utilization incidence of almost all MNCH related services is progressive in Pakistan at the national level and in all provinces with the exception of Baluchistan. This implies that the utilization benefits of MNCH related public services are more equally distributed than income suggesting that investment in MNCH related public health services can help reduce income inequality in Pakistan.

### Policy implications

On the basis of the key findings summarized above, we recommend the following policies:

#### Increase public spending on health and on MNCH in particular

The overall budgetary allocations for health in Pakistan are quite low compared to other developing countries that have a development and a public health profile that is similar to Pakistan. As percentage of GDP, Pakistan spent around 0.6 % of its GDP on health in 2010–11 [5, 21], compared to 1.2 % in India and 1.3 % in Bangladesh for the same year [2]. This is despite the fact that poverty reduction has been an important goal of the successive governments in Pakistan and public spending on health has been recognized by the government as pro-poor and has been monitored and reported under the Poverty Reduction Strategy Papers initiated by the International Monetary Fund [21].

Of the total budgetary allocations on health in Pakistan, the share of MNCH is merely 0.2 % [21]. This share needs to be increased significantly especially in light of the evidence obtained by the present study regarding the progressivity of investment in MNCH related public services.

#### Integrate MNCH programmes into income support programmes

Giving the evidence on the progressivity of public investment in MNCH, it may also be a good idea to integrate MNCH programmes with the ongoing income support programmes for the poor in Pakistan. This can potentially increase the effectiveness of both income support as well as MNCH programmes.

The Benazir Income Support Programme (BISP) is one of the largest social protection initiatives in Pakistan [22]. This programme provides unconditional cash transfers to women below a specified level of income. Although there have been some attempts to include social health protection schemes under the umbrella of BISP,^5^ it is recommended that cash transfers be made conditional on meeting certain health related goals, particularly those related to mothers and infants such as getting them immunized against certain diseases and spending a certain proportion on their health. A portion of these cash transfers can also be converted into food vouchers consisting of nutritional food items for mothers and children.

#### Expand basic health services and facilities

A key finding of the study, as discussed in section 4.1 above, is that the usage of basic health services such as immunization; prenatal consultation especially through Lady Health Workers (LHWs) and Lady Health Visitors (LHVs); and Basic Health Units (BHUs) is either pro-poor or proportionately distributed. In light of this evidence, one may propose that if the government wants to use public spending on health as a tool to reduce poverty and protect the vulnerable groups, it needs to target whatever meager amount it allocates on health towards basic and preventive health care particularly by strengthening the Basic Health Units as well as the health services provided through LHVs and LHWs while continuing to expand on immunization services.

The present budgetary allocations in Pakistan show that more than 70 % of the health sector budget is spent on specialized hospital care - that primarily benefits relatively high income groups as shown by international evidence [20] and supported by our study - and only 20 % is spent on “Health Facilities and Preventive Measures” [21]. This category includes primary health care facilities such as rural health centres, Basic Health Units, dispensaries, first aid posts, mother and child health centres, programmes such as Lady Health Worker Programme; Malaria Control Programme; Tuberculosis and HIV/AIDS Control Programme; National Maternal and Child Health Programme; the Expanded Programme on Immunisation; Food and Nutrition Programme [23]. Increasing budgetary allocations on this category is likely to enhance both the efficiency of public health spending in reducing mortality rates (since infectious diseases and maternal and perinatal conditions dominate the major causes of mortality)^6^ as well as equity in public health spending (as these services are mostly used by the poor as shown by our study).

Basic Health Units need to be expanded across rural and urban areas as their usage has clearly been found to be pro-poor. These Units may also be equipped further to provide basic reproductive health facilities such as skilled birth attendance and emergency obstetric care.

#### Improve targeting of hospital based maternal services

Since hospital based maternal delivery is found to be pro-rich in our study, it is important that public spending on these services be targeted effectively towards the poor. This can be accomplished, for example, through a voucher scheme that provides free or subsidized treatment to women below the poverty line. The access cost for hospital based services for the poor may also be reduced by locating some public hospitals in or around rural areas where majority of the poor reside.^7^ This can reduce the transportation cost for the poor who usually have to travel long distance to reach hospitals that are mostly located in urban centres.

#### Expand public health services and target them effectively towards the poor in Baluchistan

Our findings indicate that the usage of most public health services in Baluchistan is pro-rich. This could be due to the low outreach of public health services in the province and greater transportation costs involved in accessing health facilities that are few and far between (Baluchistan is the largest province in terms of area and has low population density). The empirical evidence for the low outreach of health facilities in Baluchistan is contained in Pakistan Social and Living Standards Measurement Survey (PSLM). For the treatment of malaria for instance, the survey asks the respondents as to why they did not consult the public health facility. Our analysis of 2007–08 PSLM data shows that 47 % of the households interviewed in Baluchistan replied that there were no public health care services. The lack of public health care services can be addressed by increasing the overall budgetary allocations for public health services in the province and spreading these services out across rural and urban areas.

Historically, the regional distribution of resources in Pakistan from centre to provinces has been based upon the sole criterion of population with Baluchistan receiving the lowest and Punjab receiving the highest amount. Table 9, for instance, shows the provincial shares in total health expenditure in 2007–08. These shares are roughly concomitant to the respective population shares of the provinces and are not based upon the health needs as measured by provincial health indicators.

**Table 9:** Regional allocation of total health expenditure in Pakistan by Provinces (2007–08)

Regions	% Share in total health expenditure	% share in total population
Punjab	52.13	57 %
Sindh	22.32	24 %
KP	14.57	14 %
Baluchistan	5.41	5 %
Islamabad Capital Authority (ICT)	2.24	-
Unregionalised	3.33	-
Total	100.00	100 %

Source: National Health Accounts (NHA) for Pakistan 2007-08

The recent enactment of 18th Amendment to the constitution of Pakistan in 2011 that has resulted in the devolution of several ministries including health from centre to the provinces [18], is an encouraging step in this direction. Federal tax revenues are now transferred to the provinces through a National Finance Commission (NFC) award that relies on a much more equitable distribution formula based upon poverty, inverse population density, and revenue generation in addition to population in each province [18, 19]. This distribution criterion, although much more equitable than that followed earlier, has not resulted in a significant shift in the regional distribution of resources as population is still weighted at 82 %, poverty at 10.3 %, revenue generation at 5 % and inverse population density at 2.7 % [19]. Baluchistan has one of the worst health indicators,^8^ and on the basis of equity, more resources need to be allocated for health services in the province.

### Limitations of the study

There are two important caveats that must be kept in mind while interpreting the results of this study. *First*, benefits are measured in terms of utilization of services and anyone who uses a public health service or a facility is considered as a beneficiary. The underlying assumption here is that all beneficiaries receive the same benefit. In reality, it is quite possible that the same level of health service utilization may yield different benefits to different people depending upon their medical and socioeconomic conditions. A person who is very sick or poor, for instance, is likely to get more benefit from using a particular health facility than a person who is not very sick and has a relatively better socioeconomic status.

*Second*, data on the utilization of health services do not capture differences in the quality of service provided. These differences can be substantial across rural urban and provincial divides as well as across socio-economic groups.

### A way forward: future directions for research

By highlighting some critical issues in the utilization incidence of public services for MNCH in Pakistan, the present study can be seen as paving way for future research in this area. It brings up some questions that may be addressed in future studies. For instance, now that we know that utilization benefits from some public health services in Pakistan are unequally distributed in favour of high income groups, as a next step, it would be worthwhile to investigate the major factors that lie behind such a pro-rich pattern. Is it because of poor health seeking behaviour in general among low income households (that may be due to low literacy and awareness and higher opportunity cost of seeking care)? Is it because poor income groups prefer private health care services to public services? Or is it because of low availability of health care services in regions where majority of the poor reside? These are important questions, the answers to which can be sought through a decomposition analysis of the factors contributing to inequality in the distribution of benefits from public health subsidies on MNCH in Pakistan.

## Conclusions

Public sector investments in health are considered by the government as pro-poor interventions aimed at reducing poverty in Pakistan. By focussing on the most vulnerable group comprising of mothers, infants, and children, this study provides empirical evidence that public investments in health care services for this particular group are indeed progressive in the sense that they are distributed more equally than consumption and can potentially reduce income inequality and poverty in Pakistan. However, this potential is not being tapped fully as public expenditures on health in general, and on MNCH in particular remain low. Moreover, a major chunk of budgetary allocations on health is directed towards specialized hospital care while leaving little for basic health and preventive programmes. This study provides evidence that the utilization of services that fall within the purview of basic and preventive health services such as immunization; prenatal consultation provided through lady health workers and visitors; as well as the utilization of Basic Health Units (BHUs) is either equitably distributed or pro-poor. It is therefore recommended that the government realigns its budgetary priorities towards the expansion of basic health care programmes. This is not to deny the significance of hospital based specialized services but to perhaps suggest a greater involvement by the private sector sector in the provision of these specialized services while providing direct medical subsidies to the poor in accessing them.

The provincial analysis carried out by the study reveals that the province of Baluchistan depicts an unusually high level of inequity in the distribution of utilization benefits from almost all public health services. This finding implies that the province needs to devise a strategy on how best to mitigate these inequities in health care usage. We recommend increasing the overall investment in health in the province so as to increase the outreach of health facilities in the province and targeting them effectively towards the poor by mitigating various access costs that prevent them from using public health services.

## Additional file

Additionnal file 1: Concentration Curves and Lorenz Curves for the Utilization of MNCH related Public Services in Pakistan. (PDF 510 kb)

## Footnotes

1 A quintile refers to five equal parts into which a statistical sample can be divided. In case of income distribution it refers to five income groups ranging from lowest 20 % to highest 20 %.

2 Seventy two percent of the total health expenditures in Pakistan are financed by private sources out of which 92 % consist of out of pocket expenditures (Government of Pakistan 2008).

3 Around fifty four per cent of the households interviewed by the study, conducted by the Planning Commission, Government of Pakistan (2007), reported that they were vulnerable to health care expenditure shocks.

4 The reason for these two surveys having separate names is that prior to 1998, the two surveys were conducted separately and were not integrated with each other. However in 1998-99, HIES was integrated with PSLM (that was called Pakistan Integrated Household Survey then) for the first time and since then, HIES has been conducted as an integrated survey with PSLM.

5 See e.g www.bisp.gov.pk/hi_features.aspx.

6 See e.g. National Institute of Population Studies, 2008.

7 Poverty in most South Asian countries, including Pakistan, is a rural phenomenon. Majority of the poor in Pakistan live in rural areas [24].

8 According to Pakistan Social and Living Standards Measurement Survey (2007-08), infant mortality rate is the highest in Baluchistan. Pakistan Demographic and Health Survey (2006-07) indicates that maternal mortality ratio in Baluchistan is 785 per 100,000 which is significantly higher than other provinces [5].
