# Supplementary material for: Equity in the use of public services for mother and newborn child health care in Pakistan: a utilization incidence analysis
Source: Int J Equity Health. 2016 Jul 26;15:120. doi: 10.1186/s12939-016-0405-x (PMC4962481; doi:10.1186/s12939-016-0405-x)

Appendix A

Appendix A: Concentration Curves and Lorenz Curves for the Utilization of MNCH related Public Services in Pakistan

Figure 1: Utilization of Pre-Natal Consultation in Pakistan (2010-11)

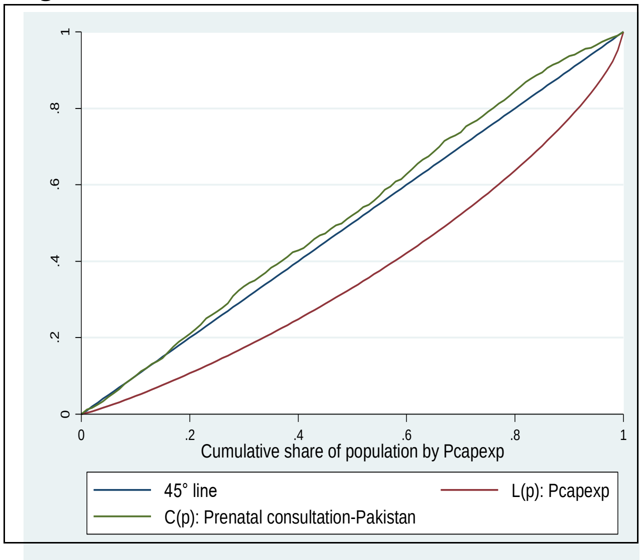

Figure 2: Utilization of Prenatal Consultation in Provinces (2010-11)

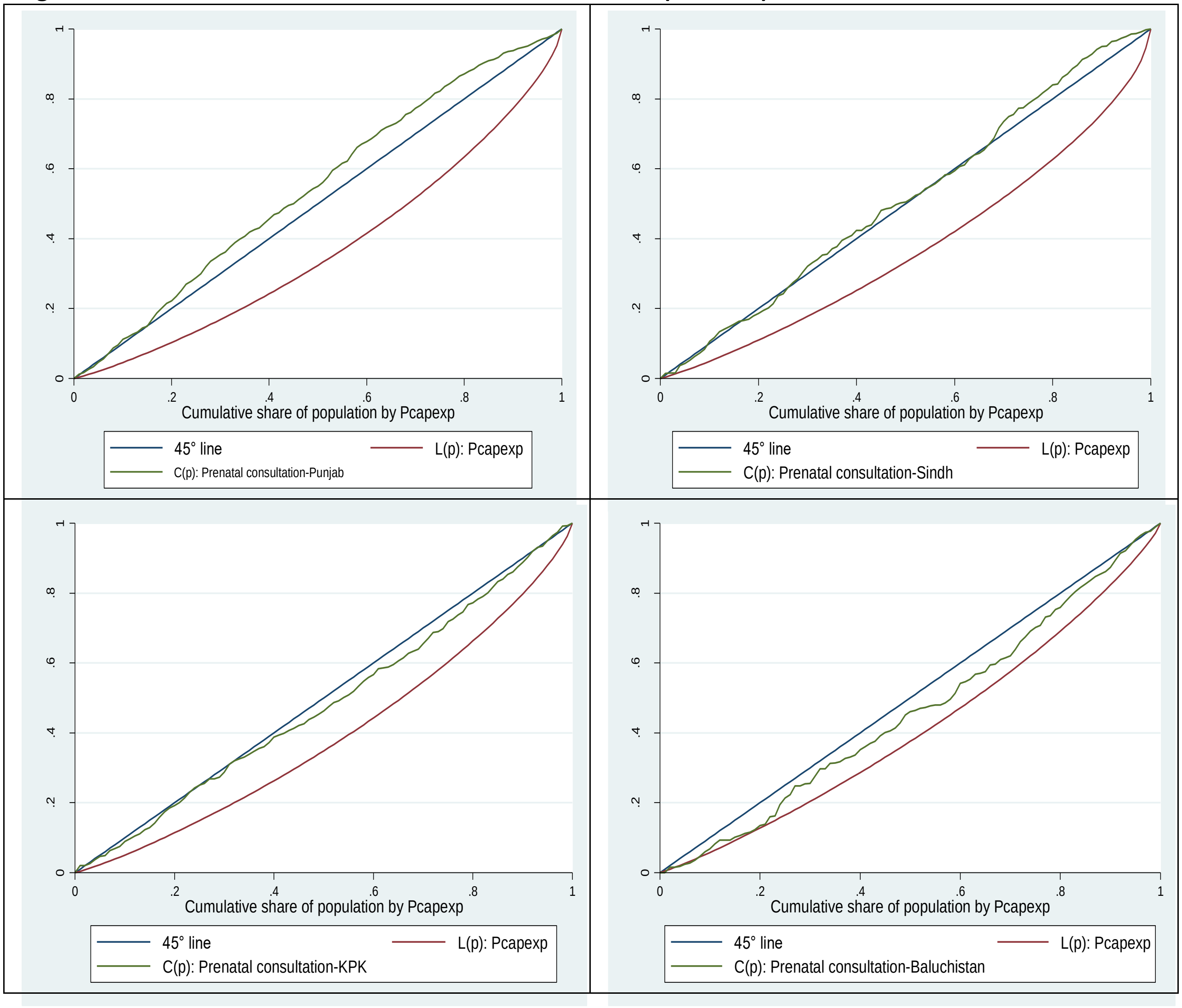

Figure 3: Utilization of Post-Natal Consultation in Pakistan (2010-11)

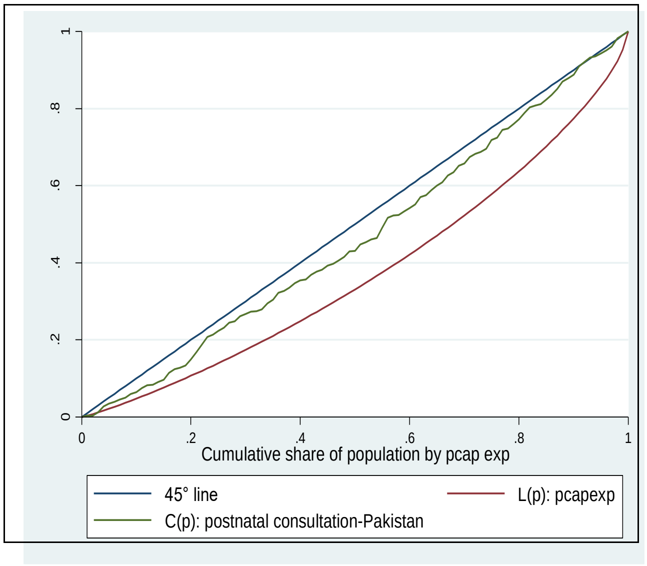

Figure 4: Utilization of Postnatal Consultation in Provinces (2010-11)

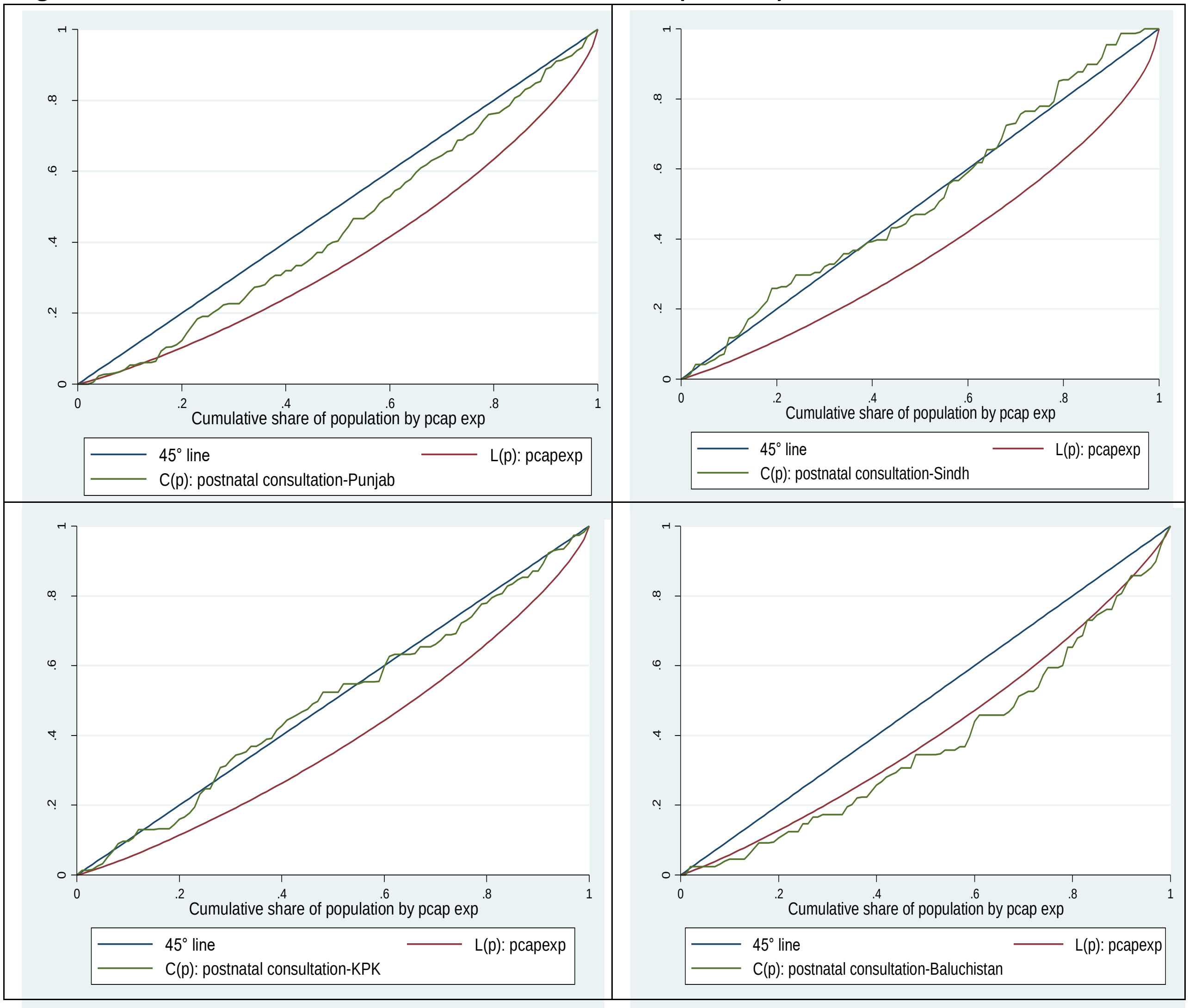

Figure 5: Utilization of Hospital based Maternal Delivery Services in Pakistan (2010-11)

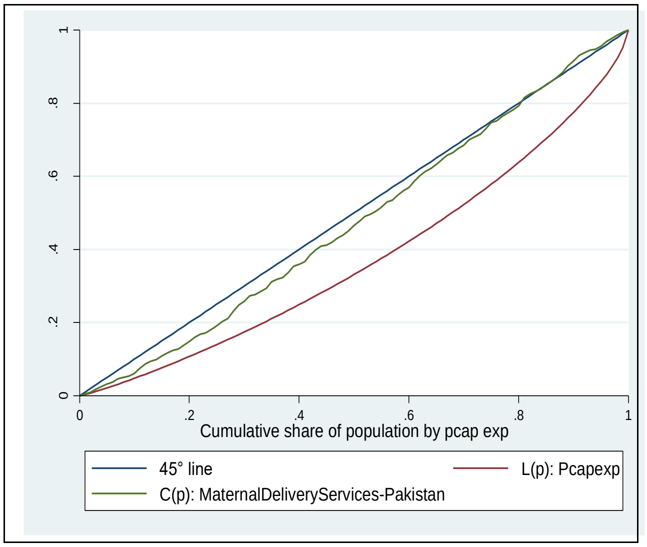

Figure 6: Utilization of Hospital based Maternal Delivery Services in Provinces (2010-11)

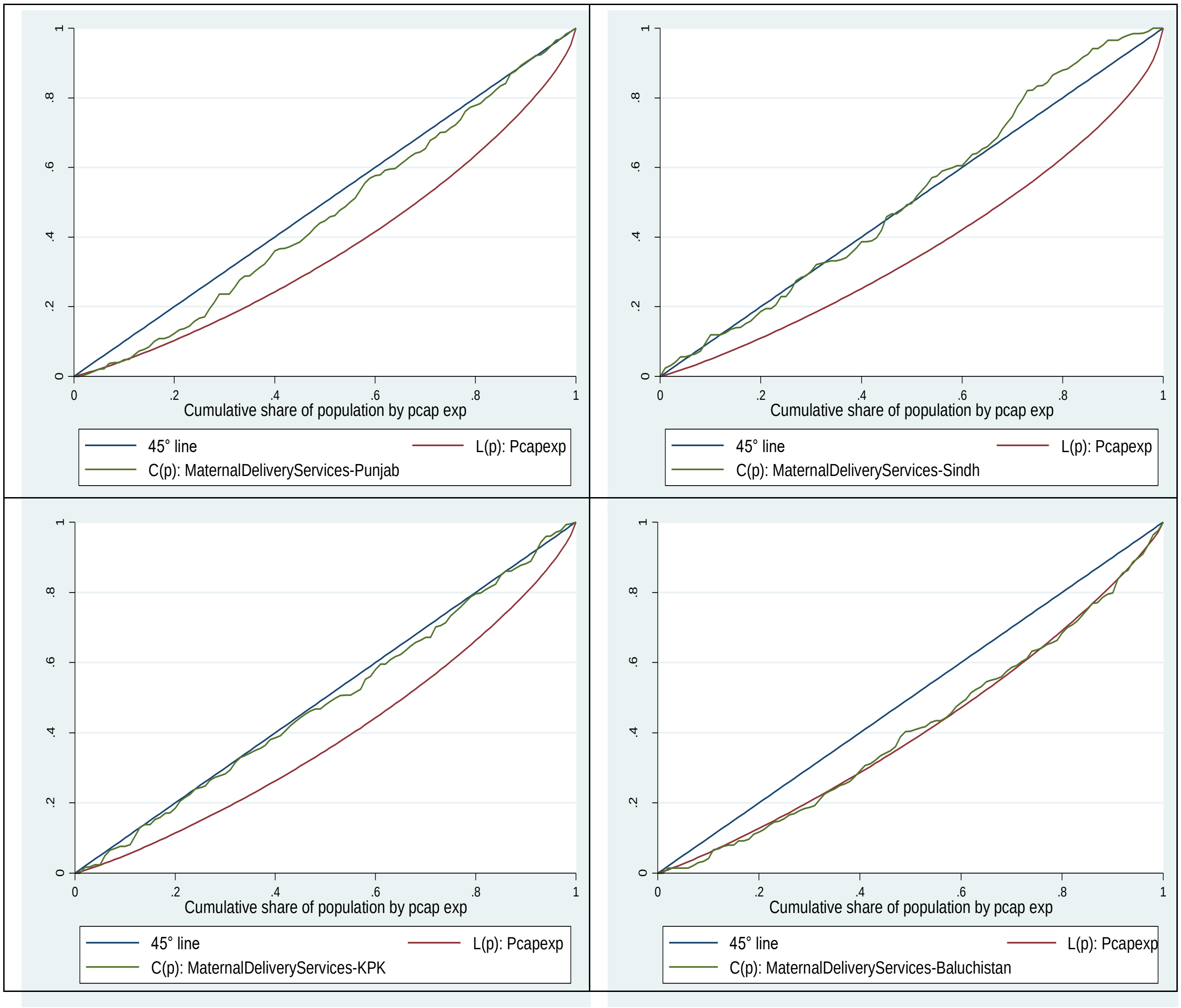

Figure 7: Utilization Incidence of Tetanus Injection in Pakistan (2010-11)

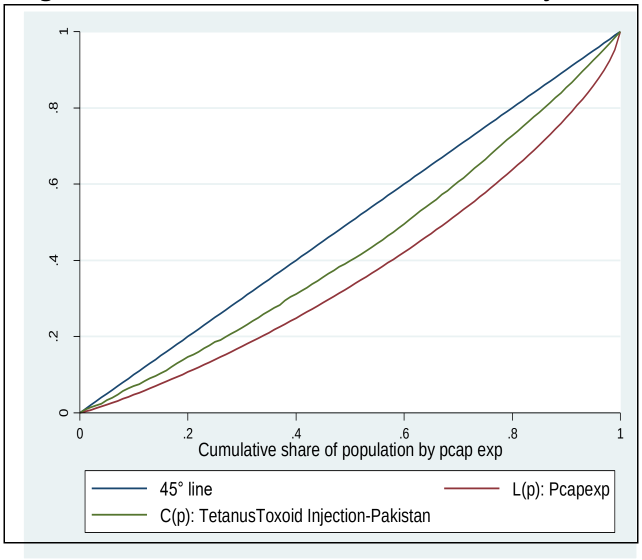

Figure 8: Utilization of Tetanus Toxoid Utilization in Provinces (2010-11)

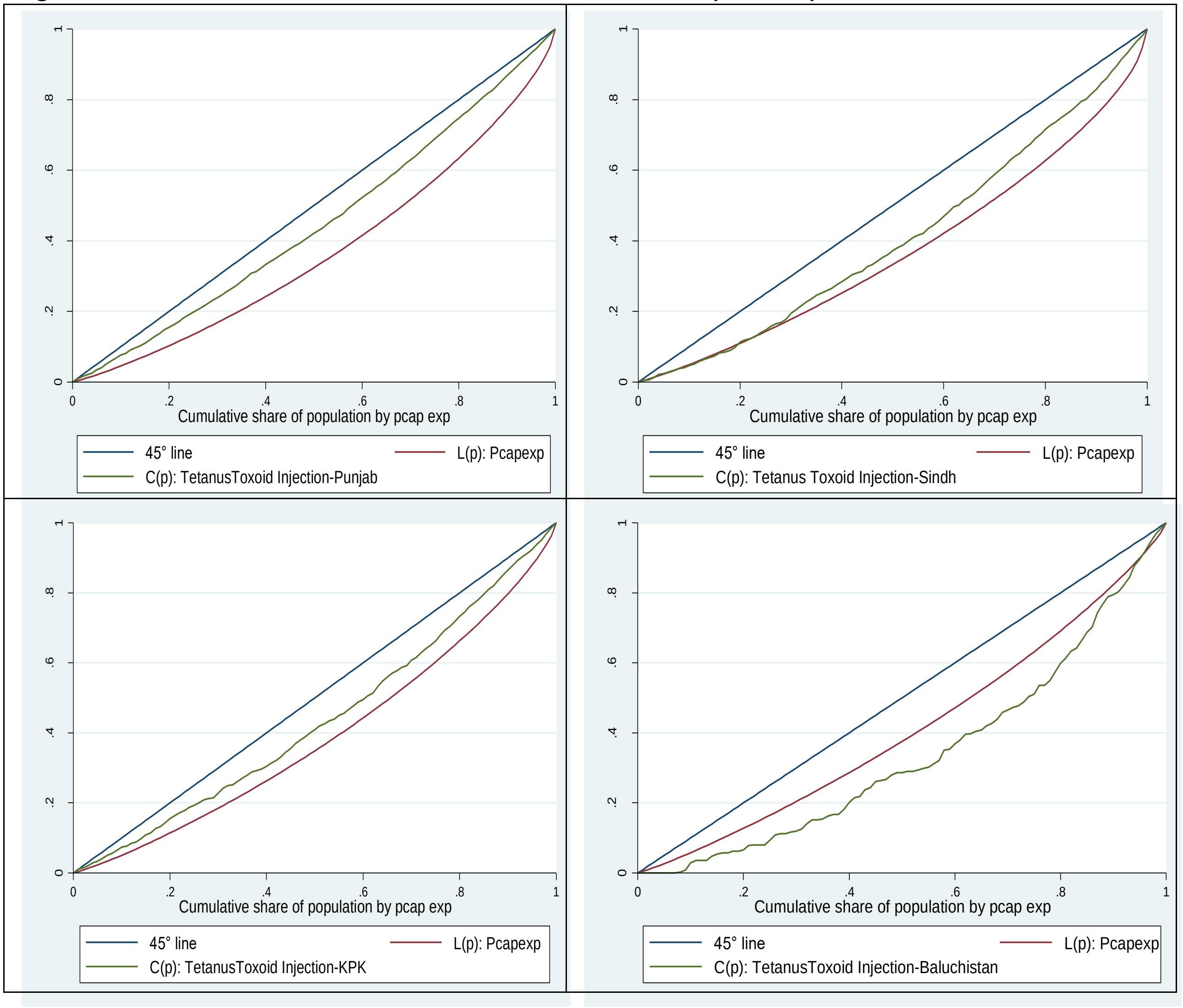

Figure 9: Utilization Incidence of Immunization in Provinces (2010-11)

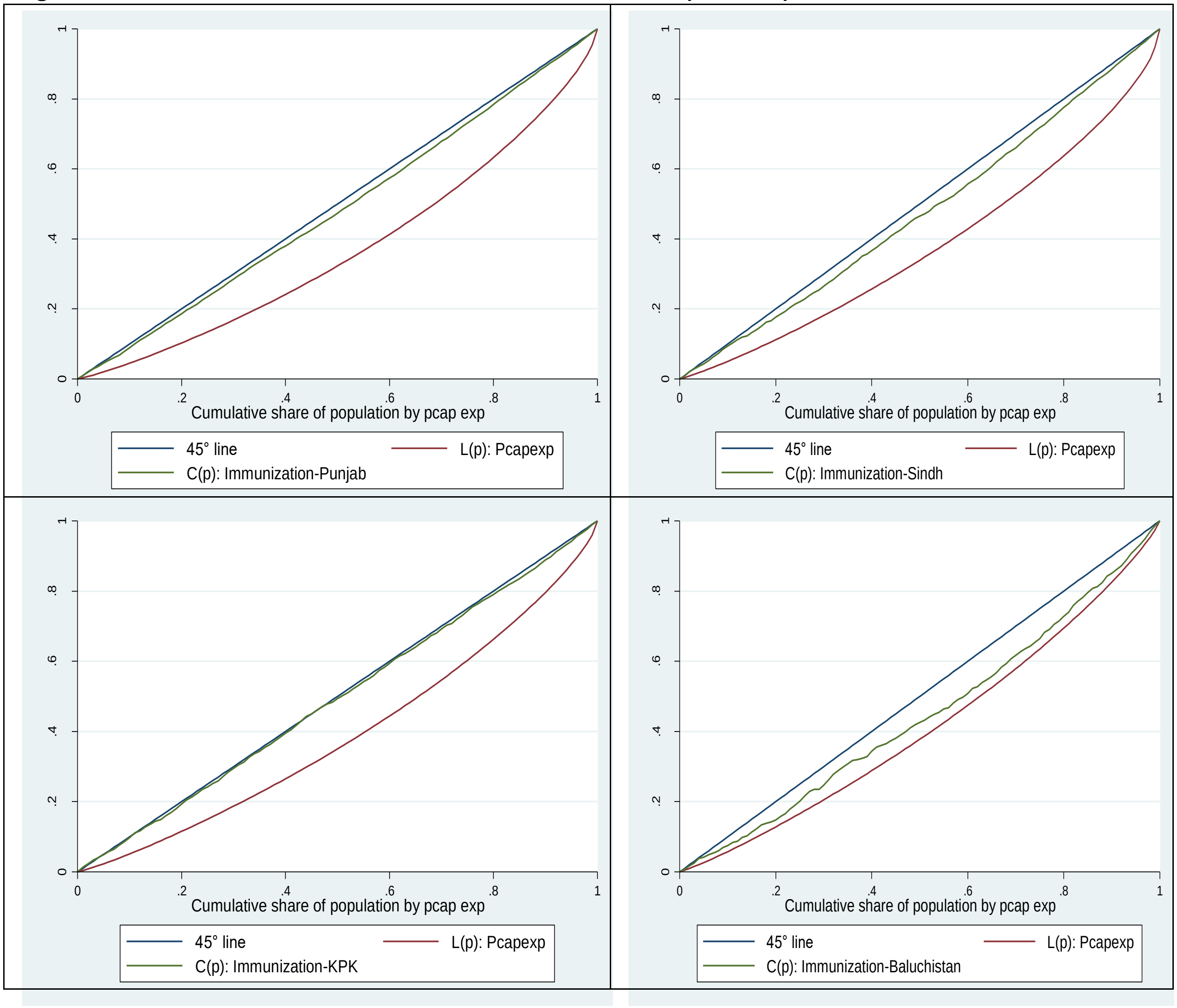

Figure 10: The Utilization Incidence of BHUs across Provinces in Pakistan

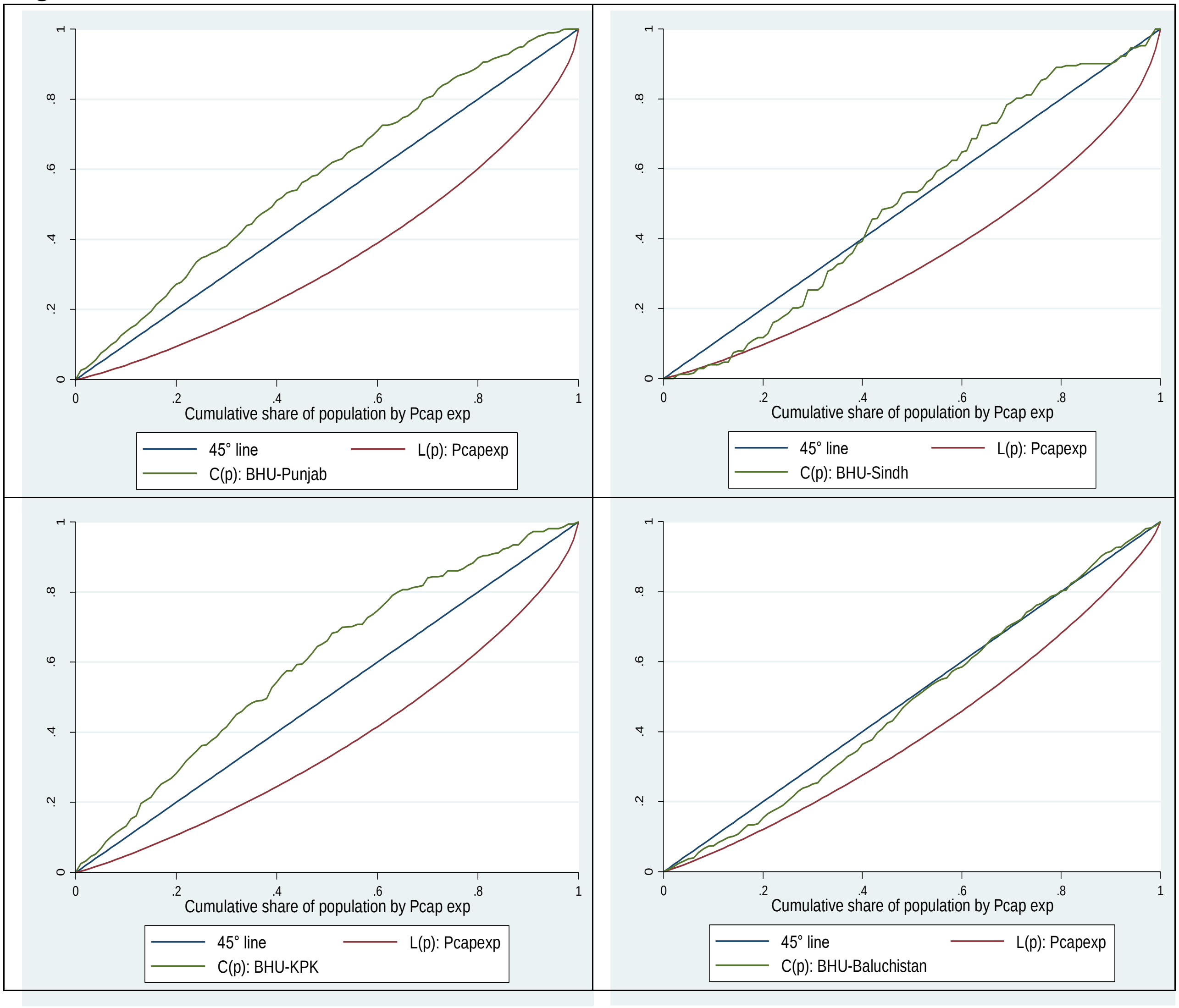

Figure 11: Utilization Incidence of Family Planning Services in Pakistan (2010-11)

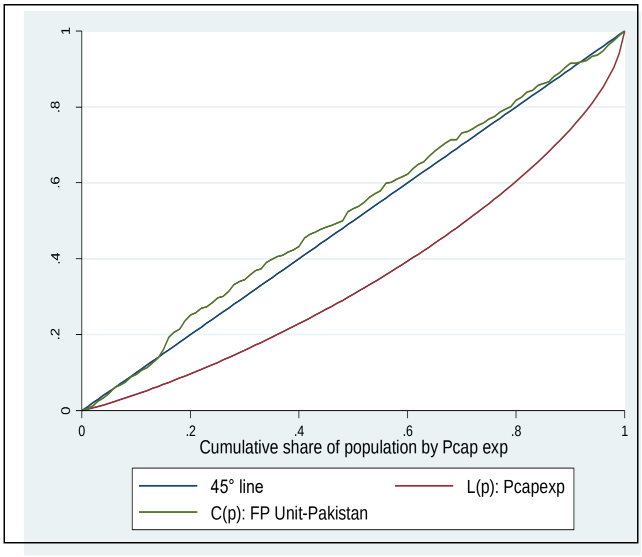

Figure 12: Utilization Incidence of Family Planning Services in Provinces (2010-11)

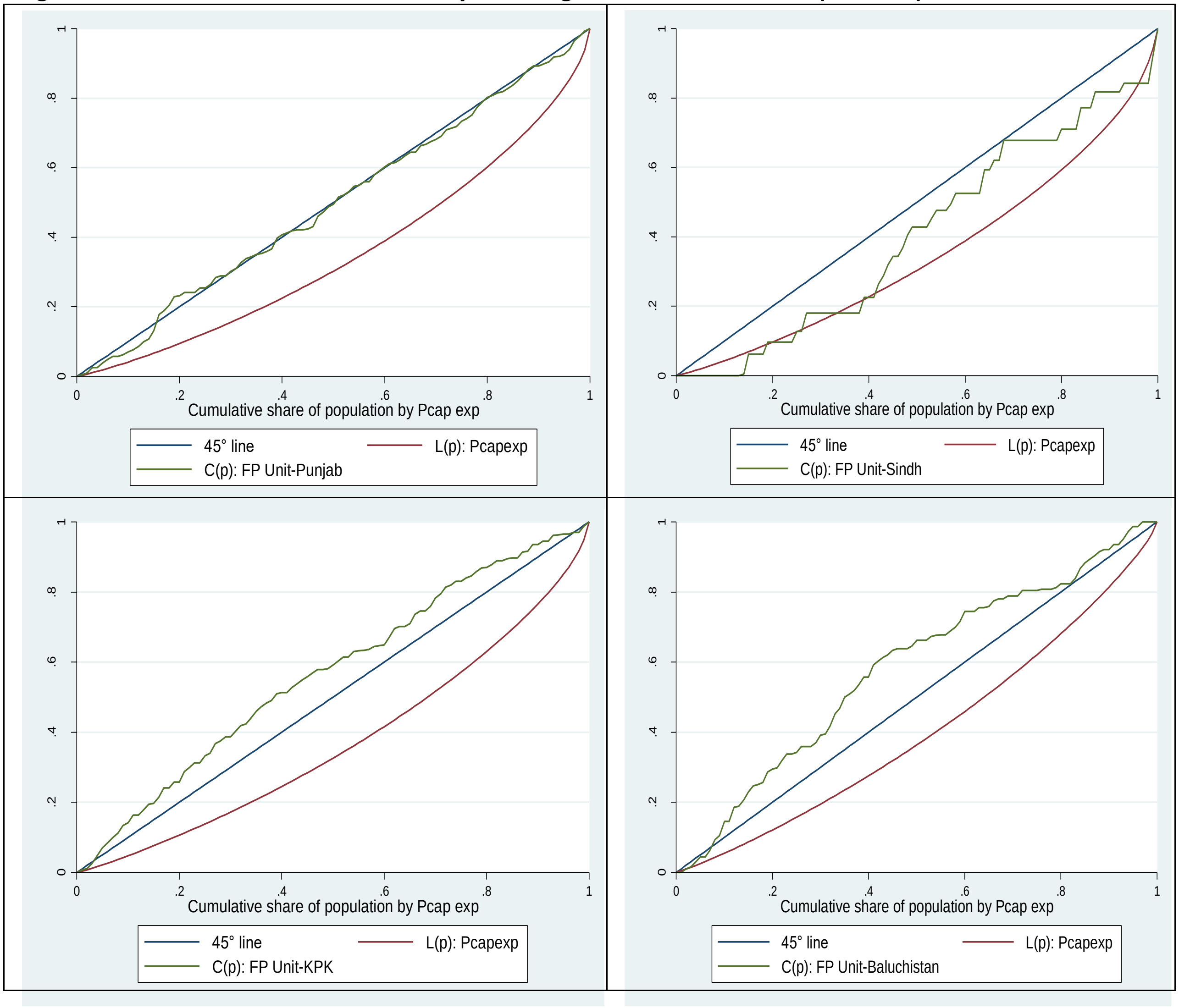

Supplement: Additionnal file 1: — Concentration Curves and Lorenz Curves for the Utilization of MNCH related Public Services in Pakistan. (PDF 510 kb) [file 12939_2016_405_MOESM1_ESM.pdf]
